# Learning from the past in the COVID-19 era: rediscovery of quarantine, previous pandemics, origin of hospitals and national healthcare systems, and ethics in medicine

**DOI:** 10.1136/postgradmedj-2020-138370

**Published:** 2020-09-09

**Authors:** Pier Paolo Bassareo, Maria Rosaria Melis, Silvia Marras, Giuseppe Calcaterra

**Affiliations:** Department of Cardiology, University College Dublin, Dublin, Ireland; Department of Biomedical Sciences, University of Cagliari, Cagliari, Italy; Department of Biomedical Sciences, University of Cagliari, Cagliari, Italy; Department of Cardiology, University of Palermo, Palermo, Italy

**Keywords:** Cardiology, congenital heart disease, paediatric cardiology, echocardiography

## Abstract

After the dramatic coronavirus outbreak at the end of 2019 in Wuhan, Hubei province, China, on 11 March 2020, a pandemic was declared by the WHO. Most countries worldwide imposed a quarantine or lockdown to their citizens, in an attempt to prevent uncontrolled infection from spreading. Historically, quarantine is the 40-day period of forced isolation to prevent the spread of an infectious disease. In this educational paper, a historical overview from the sacred temples of ancient Greece—the cradle of medicine—to modern hospitals, along with the conceive of healthcare systems, is provided. A few foods for thought as to the conflict between ethics in medicine and shortage of personnel and financial resources in the coronavirus disease 2019 era are offered as well.

*One day Albert Einstein gave his students the same final exam the year before. His assistant shyly noticed the mistake. Einstein replied: “You are right, these are the same questions as last year – but the answers have changed”*

## Introduction

In December 2019, a series of pneumonia cases of unknown origin were reported in Wuhan, Hubei province, China.

On 12 January 2020, after ascertaining the viral origin of the disease, the WHO recognised a coronavirus responsible for the disease and named it as the 2019 novel coronavirus (2019-nCoV).

Again, based on its genetic similarity to already known coronaviruses, the International Committee on Taxonomy of Viruses renamed the previously termed 2019-nCoV as severe acute respiratory syndrome coronavirus 2 (SARS-CoV-2). Conversely, the first coronavirus or coronavirus 1 was responsible for SARS epidemic in 2003.^1^

On 30 January 2020, the WHO released a public health emergency of international concern. On 11 February 2020, the WHO formally named the disease as COVID-19.

On 11 March 2020, the WHO formally recognised the global spread of COVID-19 as a pandemic: the second in this century and the first to be caused by a coronavirus.^2  3^

The first description of human coronavirus was published by the Scottish virologist June Hart Almeida in *The British Medical Journal* in 1965, while working in St Thomas’s Hospital Medical School (London). Since the spokes around the viral edge reminded researchers of a crown, the virus was named *corona* (crown in Latin) in a subsequent paper published in *Nature* (1968).^4^

Since 11 March 2020, SARS-CoV-2 has changed our daily life, behaviour and maybe the whole human history. SARS-CoV-2 has spread throughout our nightmares with the same strength as it has had in breaking into our lives.

Quarantine, spread, isolation, lockdown, cocooning, masking, social distancing, hand hygiene, blood tests, vaccine, immunity: all these words are now part of our daily speeches, pervading the most subconscious of our thoughts.

Our healthcare system is now facing huge challenges with the spread this virus, and so it is getting a favourable occasion to review the history of the previous infections which the human being had to face in the past, with the ultimate aim of educating young doctors to learn from those events.

In daily conversations, people often use the term ‘quarantine’ to refer to separating sick people with a contagious disease from the healthy ones in various ways. Quarantine or lockdown is a forced segregation to prevent a communicable disease from spreading.^5^

The term ‘quarantine’ comes from the Italian word ‘quarantena’, which means a period of 40 days (in Italian, 40 is ‘quaranta’; the latter derives from the Latin word quadrāgintā). During the 14th century, 40 days was the length of strict isolation required for ships suspected of carrying an infectious or contagious illness before their passengers and crew were allowed to land. This practice was usual in Venice in the 1300s, in an effort to stave off plague (‘black death’). It entered English language in the early 1600s.^6  7^

An early mention of ‘isolation’ dates back to the 7th century B.C. or maybe earlier in the Leviticus, the third book of the Old Testament in the Bible. In that book, the procedure of separating infected from healthy people to prevent leprosy from spreading, according to Mosaic Law was described, (if the shiny spot on the skin is white but does not appear to be more than skin deep and the hair in it has not turned white, the priest is to isolate the affected person for seven days. On the seventh day the priest is to examine him, and if he sees that the sore is unchanged and has not spread in the skin, he is to isolate him for another seven days).^8^

However, nowhere in these forerunner reports was the term ‘quarantine’ used. After arriving in Southern Europe in 1347, plague spread rapidly and reached England, Germany and Russia by 1350.^6^ Over these years, one-third of the European population passed away. The dramatic impact of the epidemic led governments to put in place extreme infection control measures. For instance, in 1374, Viscount Bernabo of Reggio, Italy, declared that every person with plague was to be taken out of that city into the fields, where to die or to recover.^9^

## Origin of the Name

The date, 27 July 1377, is one of the highest achievements in the history of medicine. Originally, the period of isolation was limited to 30 days. As reported in the first available documents, before entering the seaside city-state of Ragusa in Dalmatia (now Dubrovnik in Croatia), newly arrived people had to spend 30 days (a ‘trentine’) in a restricted place in the islands in front of the city (see box 1),^10–12^ waiting to see whether the symptoms of black death would develop. The city chief doctor Jacob of Padua was responsible for the decision. At that time, Ragusa was an important Adriatic harbour belonging to the maritime Republic of Venice.^10^ A specific decree (*Veniens de locis* pestiferis non intret *Ragusium nel* districtum) was published in Dubrovnik’s book of laws, the so-called Green Book (Latin: *Liber viridis*). The 30-day period outlined in the 1377 ‘quarantine’ edict was known in Italian as a *‘*trentino’ (‘thirty’ in English). Four were the outlined point in the 1377 edicts, namely: (1) those coming from black death-endemic regions would not be admitted into Ragusa until they had waited in isolation for 30 days; (2) none from Ragusa was allowed to access the isolation area, under penalty of being compelled to stay there for 30 days; (3) subjects not indicated by the Great Council as responsible of those being isolated were not allowed to bring them food, under penalty of remaining with them for 30 days; and (4) whoever did not accomplish with the above stated regulations would be ﬁned and subjected to isolation for 30.

In 1397, the Great Council of Ragusa released a new decree *(De ordinibus contra eos qui veniunt de locis pestiferis anno 1397 factis)*, which specified again the 30-day duration of quarantine and determined the place. Penalties were imposed for those breaking rules, and three healthcare officers (called kacamorti) were appointed to supervise the compliance with quarantine provisions. The penalties for not complying with the provided regulations were represented by a money fine or prison sentence or severe corporal punishment. The penalties were applied only to common people but not to aristocracy. In addition, the importation of goods from the countryside for the entire duration of the epidemic was forbidden.^13^ In 1448, the Venetian Senate, the main deliberative and legislative body of the Republic of Venice, prolonged the waiting period to 40 days, thus giving birth to the term ‘quarantine’.^14^ Over the next 80 years, similar edicts were published in Marseilles, Venice, Pisa and Genoa.^15^

Box 1 **Origin of the term ‘lazaret’**

- All merchants, sailors and goods coming from ‘suspicious lands’ could not enter Ragusa, unless they have spent a month on quarantine on uninhabited islands of Mrkan, Bobara and Supetar in front of the city. A few wooden shelters (wooden so that they could be burned if needed) were built on them because of the unfavourable weather. This sort of simple dwelling was called lazaret (Italian: lazzaretto). The term derives from the Biblical story of beggar Lazarus, who was raised from the dead out of the tomb by Jesus Christ. Following that, another lazaret was built on a small island in the lagoon in front of Venice in 1403. In 1467, the maritime Republic of Genoa followed the example of Venice, and in 1476, the old leper hospital in Marseille was turned into a black death hospital. The great lazaret of Marseille, perhaps the most complete of its kind, was built in 1526 on the island of Pomègues. The procedures in the lazarets in the Mediterranean sea did not differ a lot from those of the British Empire in the Levantine and North African trade. As soon as cholera arrived (1831), a few new lazarets were built, especially near the harbour of BordeauxIn the human history, similar isolation centres were built for those with leprosy (leprosarium) or tuberculosis (tubercolosarium).

Since disease was considered as a divine punishment for sinners, the biblical 40-day period of purification had crossed over into health practices and the term ‘quarantine’ had great symbolic and religious significance to medieval Christians. In the sacred scripture, when God flooded the Earth, it rained over 40 days and 40 nights; Moses stayed on Mt. Sinai for 40 days; Jesus fasted and was tempted by the devil in the desert for 40 days; after childbirth, a new mother was expected to rest for 40 days.^6^

**Figure 1:**
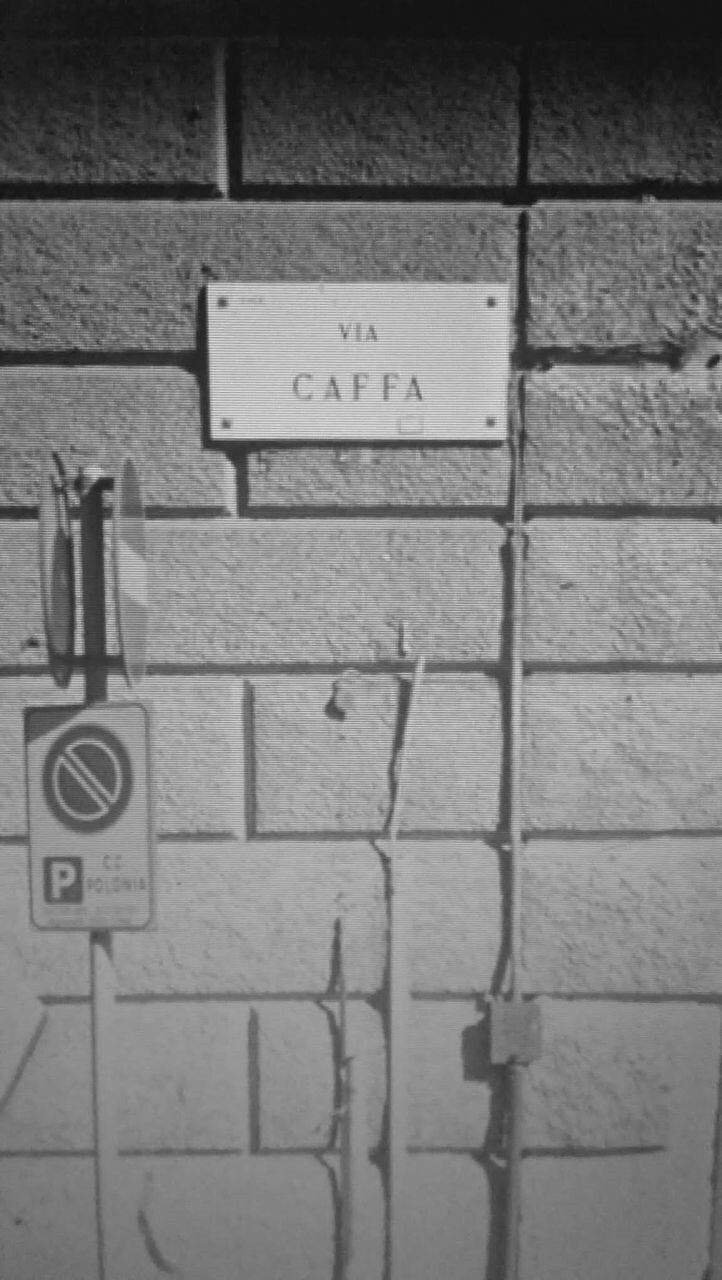
Stone plate with the name of Caffa street (via Caffa) in Genoa, Italy (author: Silvia Marras).

On the basis of a 14th-century tale by the Genoese Gabriele de’ Mussi, the devasting black death came over Italy carried by Genoese sailors who sailed from Caffa/Kaffa (now Feodosija, Ukraine) to their home city (1346/1350). In fact, in 1345, the Tartar forces captained by Jani Beg Khan had laid a military operation in which his forces surrounded Caffa, in an attempt to cut off essential supplies, with the aim of compelling the Genoese to surrender and removing them from one of the cornerstones of Europe’s defence against Eastern attack. After several unsuccessful assaults due to the outbreak of the black death, Jani Beg Khan’s army catapulted infected bodies over the city walls into Caffa, using the plague to weaken the defenders. That is the first known example of biological warfare in the human history.^16^ A Caffa street (via Caffa in Italian) is still present in the city centre of Genoa to remember that event (figure 1).

However, the number of Crimean harbours under Mongol army control suggest that Caffa is unlikely to have been the only source of plague-infested ships heading to Europe. In addition, the overland caravan routes from Mongol territories might be considered responsible for infecting Europe as well.^16^

## Plague Between Literature and Microbiology

This plague was well described by one of the most famous Italian authors of the 14th century, Giovanni Boccaccio (1313–1375), in his book *The Decameron* written in Tuscan vernacular (Italian). The book is a collection of short stories told by a group of seven young women and three young men sheltering in a villa just outside Florence to escape the black death that afflicted that city. Boccaccio probably conceived his masterpiece of classical Italian Renaissance prose after the plague epidemic of 1348, which came to a standstill by 1353. The black death in the 14th century resulted in more than 50 million deaths and is considered one of the greatest public health disasters in recorded history as well as one of the most dramatic examples ever of a periodically re-emerging disease. Between 1348 and 1359, the black death killed about one-third of the European population, and a significant percentage of that in Asia.^10  17  18^

Only a few centuries later, it was discovered that plague or black death is a bacterial disease caused by the gram-negative pathogen *Yersinia pestis*, discovered in 1894 by the Swiss/French physician Alexandre Yersin and later (1944) renamed ‘Yersinia’ in his honour. It is mainly spread by infected fleas from rats.^19  20^ According to current knowledge, the bubonic plague has an averaged 37-day period from infection to death; therefore, the European quarantines would be highly successful in determining the health of crews from potential trading and supply ships.^21^ (‘When leaving his surgery on the morning of April 16, Dr. Bernard Rieux felt something soft under his foot. It was a dead rat lying in the middle of the landing.’ Albert Camus (1913–1960), French writer, in his book *The Plague*).

## Epidemics in the Human History

Following that, in the human history, quarantine was declared many times when an infectious disease threated the population of a town, region or country. The most dangerous pandemics in the human history are summarised in box 2.^22  23^

Box 2 **Short timeline of the most dangerous epidemics in the human history**

- 430 B.C.: In Athens, a pandemic happened during the *Peloponnesian War*.
- 165 A.C.: Antonine Plague. This was likely to be caused by smallpox carried by Huns during the Barbarian invasions. This plague lasted until 180 A.C. and Emperor Marcus Aurelius was probably among its victims.
- 250 A.C.: Plague of Cyprian. It was so coined to commemorate St. Cyprian the Christian, bishop of Carthage, who witnessed and described the plague. Africa and the territories of the Roman Empire were involved. Its aetiology is uncertain. There were recurring outbreaks over the next three centuries.
- 541 A.C.: Justinian Plague. First appearing in Egypt, then it spread through the Byzantine Empire and Mediterranean area. It killed about a quarter of the world population. It is believed to be caused by the bubonic plague.
- 11th century: Leprosy. Numerous leprosy-focused hospitals were set up to accommodate victims. Leprosy was often considered a God punishment for sinners, who were so isolated. Now known as Hansen’s disease, it still afflicts thousands of people and can be fatal if not treated with antibiotic therapy.
- 1334: A plague epidemic killed two-thirds of China’s inhabitants.
- 1350: The First Plague Pandemic. The ‘black death’ caused a collapse of the British feudal system.
- 1492: The Columbian Exchange. Upon Cristoforo Colombo discovered ‘the new world’, diseases such as smallpox, measles, tuberculosis and bubonic plague were transmitted to the native populations by the Spanish. Since no previous exposure, these diseases exterminated native people of America.
- 1665: The Great Plague of London and the Second Plague Pandemic. About a fifth of London population passed away.
- 1817: First Cholera Pandemic. It was the first of seven cholera pandemics over the next 150 years. Russia, Europe, the British Empire, Africa and even Japan were involved and millions died.
- 1855: The Third Plague Pandemic. Starting from China and reaching India and Hong Kong, during is scary travel claimed 15 million victims.
- 1889: Russian Flu. It was the first significant influenza pandemic. It started from Siberia and Kazakhstan, travelled to Moscow, and from there reached the rest of Europe. By the following year 1890, about 360 000 had died.
- 1918: Spanish Flu. The avian-borne influenza killed maybe up to 100 million people worldwide. It disappeared in the summer of 1919, when most of the infected had either developed immunities or died.
- 1957: Asian influenza. It started from China and from there into the rest of the world, causing from 1 to 4 million deaths, according to different estimates.
- 1981: HIV/AIDS.
- 2003: SARS.
- 2009: H1N1 influenza pandemic.

SARS, severe acute respiratory syndrome.

As a general rule, the more the human beings became civilised—leaving agrarian life, building towns, forging trade routes to connect remote regions one another and fighting wars for supremacy— the more pandemics showed up.

In the USA, the process of developing national quarantine policy required many years. Originally, as to many other matters, the single federal states were responsible for handling the influx of infectious illnesses. However, repeated outbreaks of yellow fever induced the Congress to release the National Quarantine Act in 1878, thus creating the premises for federal involvement. In 1892, a cholera outbreak led officials giving the federal government more authority to impose the requirements. By 1921, the quarantine system was totally nationalised.^24^

According to the US Centres for Disease Control and Prevention, there is a marked distinction between quarantine and isolation. As to the first term, people are put in ‘quarantine*’* when they have neither signs nor symptoms, as they are not infected, but have been or may have been exposed to a communicable disease. This can help preventing the disease from spreading. Conversely, people are put in ‘isolation*’* when they are symptomatic or are reasonably believed to be infected with a communicable disease. This is to totally separate them from those who are not infected. Isolation may be voluntary or imposed by a federal, state or local public health order.^25^

The history of quarantine in the USA is summarised in box 3.^26^

Box 3 **Summary of the history of quarantine in the USA**

- 1799: In the harbour of Philadelphia, the first quarantine station was built after a previous yellow fever outbreak in 1793.
- 1878: Release of the National Quarantine Act, which shifted quarantine power from single states to federal government.
- 1944: The federal government quarantine authority was set up.
- 1967: The National Communicable Disease Centre replaced the federal government quarantine authority.
- 1970s: The number of quarantine stations was reduced from 55 to 8.
- 2004–2007: The number of quarantine stations was increased from 8 to 20, owing to the SARS outbreak in 2003.

SARS, severe acute respiratory syndrome.

## Brief History of Hospitals and Healthcare Systems

COVID-19 widespread is putting healthcare systems under significant pressure worldwide. A large number of patients are needing to be admitted to hospitals at the same time for intensive care. Available beds, ventilators and personnel are often not enough to face this ongoing emergency.

The first facilities for sick people were not hospitals but temples dedicated to ‘healing gods’, such as Imhotep for ancient Egyptian, Asclepius for the Greek and Aesculapius in ancient Rome. At that time, prayers, sacrifices and dream interpretations played a crucial role in the healing pathway, but the ancient doctors also stitched cuts, set broken bones and administered opium for pain.^27^

The foundations of medicine are in the island of Delos, which belongs to the Cyclades archipelago in the Greek sea. According to local mythology, Delos was the birthplace of Apollo, who is acknowledged as the original source of health and healing. He taught the art of surgery to Chiron, the centaur, who in turn taught the same to Asclepius, the Greek healing God: his daughters were Hygieia (‘Hygiene’, the goddess of cleanliness), Iaso (the goddess of recovery from illness), Aceso (the goddess of the healing process), Aegle (the goddess of good health) and Panacea (the goddess of universal remedy). Religious worship of Asclepius was in the island of Kos, which was also the birthplace of Hippocrates, who is recognised as the father of ‘rational’ medicine. In Athens, the temple dedicated to Asclepius (5th century B.C.) had a large room for inpatient to be healed (pure in the body and spirit). Greek physicians treated patients on the basis of house calls as well, which was a practice that continued for hundreds of years. The temples became medical practice schools in Greece in the 6th century B.C.^28^

The word ‘hospital’ derives from the Latin word ‘hospes’ for host or ‘hospitium’ for a place to entertain. The Roman military hospitals were often used as healthcare facilities. Each room was provided with three beds, thus representing a kind of ancient ward. Similar hospitals were likely to be available also for gladiators and slaves, owing to their financial value. However, public hospitals were not at patients’ disposal, and physicians examined them at home after being called.^29^

Claudius Galenus, who was born in Greece and then moved to Rome, was initially a surgeon for gladiators (coerusicus) and after that a physician and a philosopher. He became the Emperor Marcus Aurelius’ personal doctor and wrote about the plague, which afflicted Rome during his reign. Since Marcus Aurelius’ family name was Antonine, the pandemic disease was called ‘Antonine Plague’. Galenus contributed widely to the progress in medicine and his books were considered fundamental up the Middle Ages.^30^

As the Roman Empire got converted to Christianity, the Church’s role in providing for the sick persons became firmly established. Many monasteries were built, and generally they included rooms for pilgrims, the poor and the sick.^31^

The emperors from the 6th century onward, including Charlemagne, decided that a hospital should be built beside every cathedral and provided with large, open wards. In the meantime, doctors kept on making house calls to the upper class. The strong religious influence in early healthcare is testified by the duties of the Warden of St Mary’s Hospital in England in 1390. Not only he was required to guarantee the quality of the care but also to hear the confession of the patient before admission (‘pure in the body and spirit’).^32^

The wards, housing multiple patients, kept on being expanded and became the standard of care for public hospitals over centuries. The cross-shaped plan, which is thought to have had origin in Florence (Italy) during the Italian Renaissance, was conceived with an altar in the middle and multiple wards radiating from it. Florence was well recognised for the high level of its hospitals and doctors, as testified by Martin Luther, during his visit.^33^

As the wards became larger, they often became the repository of infections. By the mid-1700s, one of the largest hospitals in Paris had deteriorated to awful conditions, with wards which had over 100 beds with multiple patients per bed. The wards were dark, poorly ventilated and dirty.^34^

A significant improvement was reached, thanks to Florence Nightingale, the founder of modern nursing, after seeing a mortality rate of over 42% at a military hospital in Turkey during the Crimean War. A new type of hospital, made up of pavilions rather than wards, was conceived. Pavilions provided patients with fresh air and daylight, thus improving their recovery and reducing infections. This approach is sometimes called ‘the Nightingale Ward’ and was used first in St Thomas’ Hospital in London.^35^

Even at the old religious hospitals, nobles could have better housing bed by making donations. This approach was expanding in the late 1800s, so that in 1842, the first ‘pay’ hospital was opened in London with eight private single-bed rooms.^36^

As time passed, each country set up its arrangements for meeting and keeping people healthy, treating the sick and protecting from infectious diseases. Healthcare innovation is a challenging topic for legislators and governments worldwide. Only the most developed, industrialised countries (about 40 of the 200 in the world) have established healthcare systems, while the others are too poor for providing their citizens with any kind of mass medical care.

Healthcare system models are usually summarised in terms of three main models (or their combination), with expected different opinions in relation to which is the best of them.^37^

The first model is called *Bismarck Model*. Germany has the world’s oldest national social health insurance system, whose origin dates back to 1883 with the Otto von Bismarck’s Sickness Insurance Law. It uses an insurance system—the insurers are called ‘sickness funds’—which are usually financed jointly by employers and employees by means of payroll deduction (‘mutualistic’ system). The Bismarck Model-based system is not aimed at making profits. Doctors and hospitals tend to be private in Bismarck countries. For example, Japan has way more private hospitals than the USA. The Bismarck Model does not provide with universal health coverage, since it requires employment for health insurance and stores its resources only for those who contribute financially.^37^

The second is the *Beveridge Model*. It was developed by Sir William Beveridge in 1948 in the UK. It is a centralised system working through the establishment of a national healthcare service (in the UK, it is the National Health Service). In summary, the government acts as the single-payer, because of taking off all competition in the market to maintain costs low and standardise benefits. The national healthcare service being the single-payer controls what doctors can do and what they cannot. This model is funded only by taxes, and there are no out-of-pocket fees for patients or any cost-sharing. Everyone who is a tax-paying citizen is guaranteed the same access to care, so that this model is also defined as ‘universal’. One criticism of the Beveridge Model is its potential risk of overuse. Owing to the lack of restrictions, free access can potentially allow patients to demand healthcare services that are unneeded or wasteful. The result leads to increasing costs and taxes.^37^

The third model is the *Free Market Model*. Health is considered as a consumer good, in a free market for insurance companies aimed at making profits. It is the model adopted in the USA, where healthcare is not provided and financed by the government. Those who have no health insurance have to pay the bill out-of-pocket at the time of a treatment is provided.^37^

As told, especially in Europe, a combination of the above-stated models is possible. What is sure is the fact that, due to COVID-19 rapid widespread, all these systems are facing an enormous funding crisis so far, whose consequences are also ethical.

## Brief History of Ethics in Medicine and New Challenges on the Horizon

The word ‘deontology’ comes from the Greek word ‘deon’, which means ‘duty’. Thus, deontology is also named as duty-based ethics. In deontology, reason is used to reach an agreement as to ethical standards to follow, so one has the duty to act according to those ethical principles. Deontology is also called non-consequentialist ethics, because the rightness or wrongness (morality) of an action is judged on the basis of how it fits with ethical principles, not on its practical result.^38^ In this respect, the influence of the philosopher Emmanuel Kant is evident. According to Kant, the moral law or ‘categorical imperative’ is an ethical principle, a universal law for all people. The essence of morality is in these principles.

One of the oldest ethical code in the human history is the Hippocratic Oath. It was written by the ancient Greek physician Hippocrates (460 B.C.–370 B.C.). The traditional Hippocratic medical principle is ‘primum non nocere*’* (first of all, avoid any harm) and doing good. Its modern version, released in 1964 by Lasagna, Dean of the School of Medicine at Tufts University (USA), is still considered as a guide to conduct by the medical profession in modern ethics of deontology.^39^

Since the introduction of the current bioethical paradigm by Beauchamp and Childress in 1979, there has been much controversy about whether its four principles are universally and effectively applicable. These principles are (1) respect for autonomy, (2) justice, (3) beneficence and (4) nonmaleficence. The reported issues as to applicability are related to many factors, including the continuous changing of social structures and conditions, and the ongoing advances in technology. As to ethics, doctors have often to face other subjects who may have had no medical training, such as bioethicists, moral philosophers, theologians, clergy, hospital administrators, lawyers, attorneys, judges. Living in a country with limited economic resources is another factor sometimes hampering the practical application of these ethical obligations.^40^

Persad *et al* suggested four ethical principles to guide the distribution (allocation) of the available scarce medical resources, namely (1) treating people equally, (2) giving priority to the worst off, (3) maximising benefits and (4) promoting and rewarding social usefulness.^41^ Within each of these four principles, there are two further contrasting ethical sub-principles (for a total of eight sub-principles). For example, regarding ‘maximising benefits’, two sub-principles are provided, that is, ‘saving the most lives’ and ‘saving the most life years’.^42  43^ During COVID-19 pandemic, this is one of the new challenges faced by doctors on the front line. Since our medical systems have often only a limited amount of available resources (in terms of nurses, physicians, beds, treatments, equipment, money), doctors may have to choose whether treating a patient or another, based on his/her life expectancy.

In addition to clinical responsibilities, the COVID-19 outbreak is posing a very real ethical dilemma: what is a doctors’ responsibility to serve patients despite personal risk? Is this risk eventually to extend to medical trainees? The American Medical Association Code of Ethics states that a physician’s responsibility to provide urgent care during disaster situations holds ‘even in the face of greater than usual risk to the physicians’ own safety, health or life’.^44^

There are also other new and still unanswered questions. How can a doctor maintain a patient’s humanity during an overwhelming pandemic with limited resources and time? Is the principle of delivering ‘empathetic care’ to be redefined under the current circumstances, and, if so, what does that look like? Is sacrificing personal freedom with lockdown to protect the safety of communities justified? Is sacrificing the individual healing and dignity of patients to protect the health and welfare of the general population allowed? Should individual rights be limited in favour of supporting more vulnerable people? We are social creatures and the principle of ‘solidarity’ implies that we act supporting the most vulnerable members of our society and that we must not left them alone in scary times. In a more personal way, all of us have a friend or beloved one who is at risk in case of COVID-19. What would we want others to do to protect them?

An interesting ethical perspective is represented by the immunity-based licences recently suggested, similarly to those for drivers or aeroplane pilots. However, one may argue that the latter are a certification of ability to work, not of health or personal state. Unlicenced people should be subject to social or economic exclusion as well as personal liberty limitations. Never the current restrictions were applied before in Western societies.^45  46^

In ancient Greece, crisis had also the meaning of ‘change’. The COVID-19 outbreak found most of countries unprepared, with scarce resources stored to afford the SARS-CoV-2 emergency. Coronavirus has been dismantling our daily life in all its features (health, behaviour, economy).^47^ In a catastrophic perspective, since a second outbreak cannot be excluded, we should be ready to cope with it by building new specific COVID-19 hospitals and preparing nurses and general practitioners to face the emergency at the very early beginning, thus preventing patients from the need to be admitted to hospitals. All the above have strong ethical consequences and imply a new way to allocate resources.

There are two possible long-term strategies: ‘long-term mitigation’ and ‘suppression’. The first implies sustained or intermittent social distancing until herd immunity is reached or a vaccine discovered. Conversely, the second is aimed at identifying and isolating cases until a vaccine is available. Suppression requires dropping case numbers quickly to be effective, by using excellent testing and contact tracing infrastructure.^48^

## Conclusions

Nowadays, a new pandemic is threatening our hyper-technological, but even so defenceless world. Due to its sudden and too recent appearance, evidence-medicine on this issue is still poor to provide clinicians with reliable data.^49^

A new viral disease (the Great Invader) has surprised an unarmed world. It causes pneumonia, extrapulmonary complications and often death. The first case was reported in China on 17 November 2019 but was not recognised soon. Eight more cases appeared in early December 2019, with researchers starting to think about a still unknown virus as their possible cause, but only on 31 December, they were reported to the WHO Country Office. On 11 February 2020, the infection was officially named COVID-19. Following that, the virus spread beyond Chinese borders and, by mid-March, reached more than 163 countries. On 11 March 2020, the WHO announced that COVID-19 was officially a pandemic. The spread is anywhere near finished.^50  51^ Without a vaccine available and due to the lack of a reliable therapy, a vast majority of the countries identified persistent lockdown as the only way to limit contagion from spreading without control. Overall, the 7-century-old ‘quarantine’ word has back in fashion.

Doctors are now facing new challenges, which involve ethics and may lead to make difficult and painful choices for their patients and themselves, owing to the paucity of the available resources.^52^ The current healthcare systems are unlikely to be prepared to cope with this sudden and unexpected emergency and should be reformed or updated (from lazareth/leprosarium/tuberucolosarium to ‘COVID-sarium’, ie, a specialised centre with personnel for swabs and immunology tests).

We should be careful that the ‘cure’ for COVID-19 is not worse than the disease itself. The universal and unprecedented public health preventive measures, including lockdown and social distancing, are damaging our economy and increasing unemployment worldwide.

Lastly, as doctors, the current COVID-19 age is emphasising some of the moral problems of modern medicine, which is more than ever split between ethics and financial rationalisation.
